# Successive four-phase liquid separation using hierarchical microcube-nanohole structure and controlled surface wettability meshes

**DOI:** 10.1038/s41598-019-43003-1

**Published:** 2019-04-24

**Authors:** Seeun Woo, Woonbong Hwang

**Affiliations:** Department of Mechanical Engineering, POSTECH, Pohang, 37673 Republic of Korea

**Keywords:** Design, synthesis and processing, Design, synthesis and processing, Molecular self-assembly, Molecular self-assembly

## Abstract

The chemical industry needs filter systems with selective wetting properties for environmental protection and effective liquid separation. Current liquid-separation systems are mainly based on the surface energy of the meshes used to separate liquid particles; the smaller the difference between the surface tension of the liquids to be separated, the lower the separation efficiency of these systems. Sophisticated control of the surface wettability of a separation system is necessary to separate liquids with small differences in their surface tension. We precisely adjusted the surface-energy threshold of aluminium meshes used for separation by simply coating their hierarchical microcube and nanohole structures with different materials. We also applied patterning technology to create a single mesh with a heterogeneous distribution of surface tension to successively separate four liquids. Under the force of gravity, the hybrid system of meshes effectively separated the mixture of four liquids, yielding a perfect collection rate (≥98%) and high content ratio (≥96%). Even multiphase mixtures of immiscible liquids with surface tension differences as small as 10.4 mN/m could be effectively separated. Thus, multiphase liquid-separation systems can be used for the efficient and economical separation of various liquid mixtures in many industrial and environmental fields.

## Introduction

The operation of most liquid-separation systems is based on the difference in surface energy of the liquid components^1^. Water and oil, which show a large difference in surface energy, are relatively easy to separate. A wide range of water–oil separation systems have been developed through the use of porous materials with special wetting behaviour^2–11^. However, oil–water separation is not sufficient to meet the complex requirements in many industrial sectors, and successive separation systems for multiphase or organic liquid mixtures are needed. For example, successive separation processes are often necessary in various chemical applications such as anhydrous heterogeneous chemical reactions^12,13^, biodiesel production processes^14^ and extraction of multiple liquid phases^15^. However, because the continuous separation of multiphase liquids with small differences in surface energy is more difficult than the separation of liquids with large surface-energy differences, it has not been widely studied, and efficient execution of multiphase liquid separation remains a challenge.

To separate a multiphase liquid mixture whose components have similar surface energies, the surface of the separation system must be designed to allow accurate control of its wettability. More specifically, the filter should exhibit opposite wettability by the components in the mixture^16^. In our earlier study, we fabricated aluminium meshes that could sequentially separate three different liquids from a mixture without any external assistance^17^. However, we also discovered that a difference of 40 mN/m among components with surface energy in the range of 32.0–72.0 mN/m was too wide for efficient separation of the liquids. We believe that a novel mesh with superhydrophilicity–underwater-oleophobicity would allow successive separation of a liquid mixture with surface energy in the range of 32.0–50.8 mN/m in a simple, efficient, and economical manner. With this mesh, effective separation can be expected when the range of surface energy is reduced by half, and the difference between the surface tensions of individual components is limited to <20 mN/m. Studies have shown that a superhydrophilic material can trap water on rough surfaces, and the trapped water greatly weakens the contact between oil and the superhydrophilic material, creating a superhydrophilic–oleophobic surface with low adhesion to the mesh^18–20^.

In the follow-up to the earlier study, we added a superhydrophilic–underwater-oleophobic aluminium mesh to the multiphase liquid-separation system; this mesh could be used for successive separation of four-phase solution mixtures under gravity. The superhydrophilic–underwater-oleophobic mesh, superhydrophobic–superoleophilic mesh, and superhydrophobic–superoleophobic mesh of the system were designed to repel liquids with surface tensions (*γ*_sl_) above 50.8, above 32.0, and above 21.6 mN/m, respectively. Therefore, liquids with surface tensions of ≤21.6 mN/m, 21.6–32.0 mN/m, 32.0–50.8 mN/m and ≥50.8 mN/m could be separated from each other. Four liquids, whose surface tensions represent these target separation ranges—water (H_2_O; *γ*_sl_ = 72 mN/m), diiodomethane (CH_2_I_2_; *γ*_sl_ = 50.8 mN/m), 1,2-dichloroethane (C_2_H_4_Cl_2_; *γ*_sl_ = 27.8 mN/m), and pentane (C_5_H_12_; *γ*_sl_ = 15.8 mN/m)—were selected for this study. During the separation, diiodomethane, which was repelled by the superhydrophobic–superoleophobic, superhydrophobic–superoleophilic, and superhydrophilic–underwater-oleophobic meshes, was collected in the first beaker; water, which was repelled by the superhydrophobic–superoleophobic and superhydrophobic–superoleophilic meshes, was collected in a second beaker; 1,2-dichloroethane, which was repelled by the superhydrophobic–superoleophobic mesh, was collected in a third beaker; and pentane was collected in the fourth beaker after passing through the superhydrophobic–superoleophobic mesh. Furthermore, the meshes exhibited high separation efficiency for multiple four-phase liquid mixtures and excellent structural stability. In addition, a surface with regions of three different types of wetting was fabricated with a patterning technique to demonstrate that multiphase liquids can be separated by a single mesh. We believe that the information obtained in this study could greatly simplify the successive separation of many four-phase mixtures.

## Results

**Figure 1:**
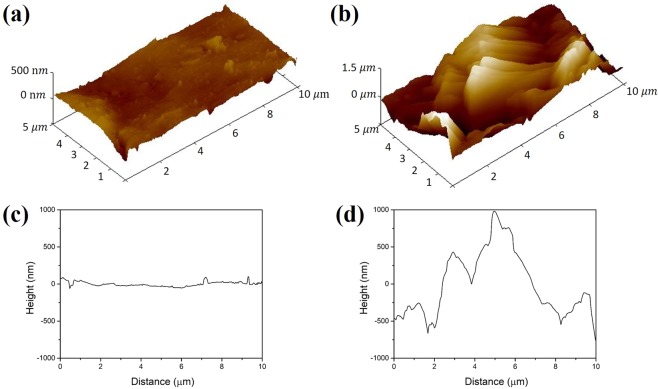
(**a**,**c**) Three-dimensional view of AFM topographic map and height profile of a pristine aluminium mesh. (**b**,**d**) Three-dimensional view of AFM topographic map and height profile of the aluminium mesh after HCl etching and anodization.

As is well known, the wettability of a surface is mainly governed by its chemical composition and roughness^21–24^. In particular, the hierarchical microscale and nanoscale structures of the meshes play important roles. The surface roughness of the microstructures formed by acid etching and anodizing of an aluminium mesh was investigated by analysing atomic force microscope (AFM) images of its surface. Figure 1 shows representative AFM three-dimensional views of the pristine and treated aluminium meshes, as well as their topographic height profiles. It can be clearly seen that the etching and anodizing processes resulted in a significant increase in the surface roughness, commonly represented by *R*_a_, the arithmetic average of the surface roughness in nanometres. The larger the value of *R*_a_, the rougher the surface. *R*_a_ of the untreated aluminium mesh was 35.3 nm, and *R*_a_ of the etched and anodized aluminium mesh was 410 nm; the resulting hierarchical microstructures and nanostructures thus favoured wetting of the surface.

**Figure 2:**
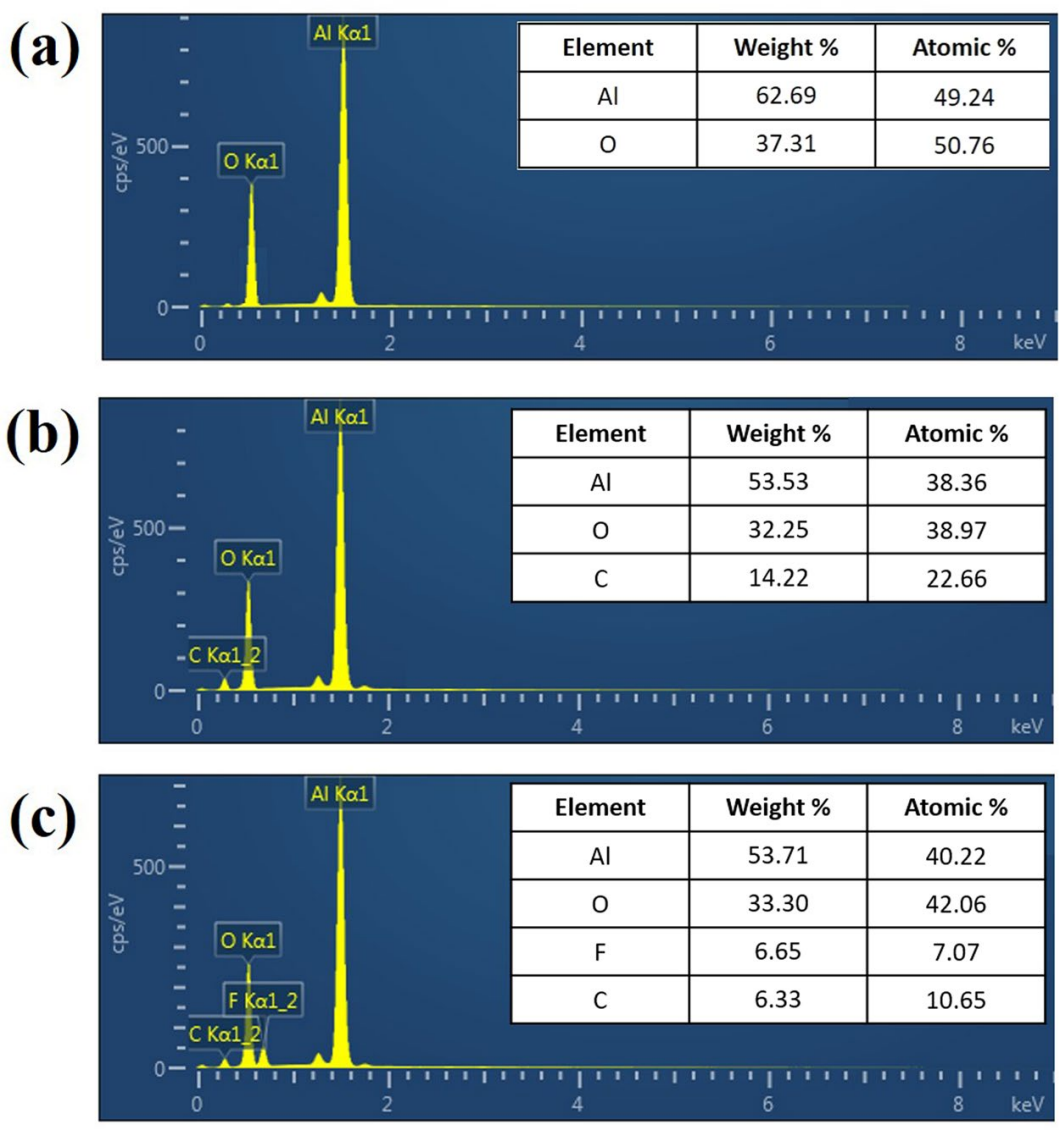
EDS analysis results for the fabricated surfaces: (**a**) uncoated underwater-oleophobic mesh; (**b**) OTS-coated superoleophilic mesh; (**c**) HDFS-coated superoleophobic mesh.

Energy-dispersive spectroscopy (EDS) was used to investigate the changes in the chemical composition of the three aluminium meshes fabricated for this study. Figure 2(a–c) show the results of EDS analysis for the underwater-oleophobic, superoleophilic, and superoleophobic aluminium meshes, respectively. The strong Al peak and O peak in Fig. 2(a) have an intensity ratio that is similar to that in the spectrum of Al_2_O_3_ crystals and corresponds to the previously reported value^25^. After an octadecyltrichlorosilane (OTS) coating was formed on the underwater-oleophobic mesh, an additional carbon peak was observed in the spectrum, as shown in Fig. 2(b). The atomic ratio of carbon increased to 22.66%. The survey spectrum shows peaks corresponding to three elements—Al, C, and O—which indicate that OTS was successfully absorbed on the surface of the aluminium mesh. Unlike the ED spectrum of the uncoated underwater-oleophobic mesh and OTS-coated superoleophilic mesh, the ED spectrum of the superoleophobic aluminium mesh surface, obtained by coating a clean aluminium mesh with heptadecafluoro-1,1,2,2,-tetrahydrodecyl trichlorosilane (HDFS), exhibits an additional fluorine peak corresponding to an atomic ratio of 7.07%.

**Figure 3:**
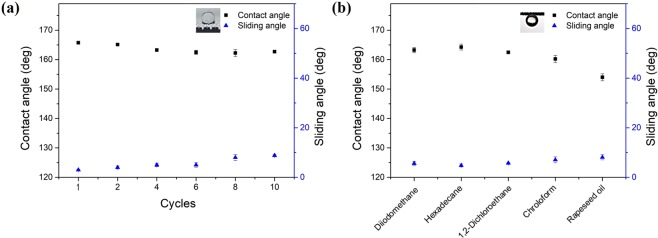
(**a**) Contact angles and sliding angles of diiodomethane (CH_2_I_2_) droplets on the mesh prepared after different coating cycles. (**b**) Underwater-oleophobic property of the mesh for a series of oils.

Our previous work proved that the fabricated hierarchical microcube–nanohole structured meshes (pore size: 300 µm) had superhydrophobic–superoleophobic and superhydrophobic–superoleophilic properties (Supplementary Fig. S1). In order to evaluate the performance of the meshes (pore size: 300 µm) in liquid separation, we further investigated their underwater wettability by oil. The wettability of the other two meshes with different pore sizes is shown in Supplementary Fig. S2. Figure 3(a) shows the wetting behaviour of diiodomethane on the mesh prepared after different coating cycles. The prepared prewetted mesh was underwater-oleophobic, with oil contact angles above 160°. In addition, the sliding angle was only 5.6° for the optimized mesh obtained after 10 coating cycles, indicating its low oil-adhesion property. The uncoated underwater-oleophobic mesh was hydrophilic in air and trapped water in the mesh. The trapped water increased the oleophobicity by reducing the contact area between the oil droplets and the mesh surface. Figure 3(b) shows the wetting of the mesh surface by various oils. The contact angles of all oil droplets were greater than 154° and the sliding angles were less than 8°. Therefore, unless specified otherwise, the best as-prepared prewetted underwater-oleophobic mesh was used in the subsequent experiments.

**Figure 4:**
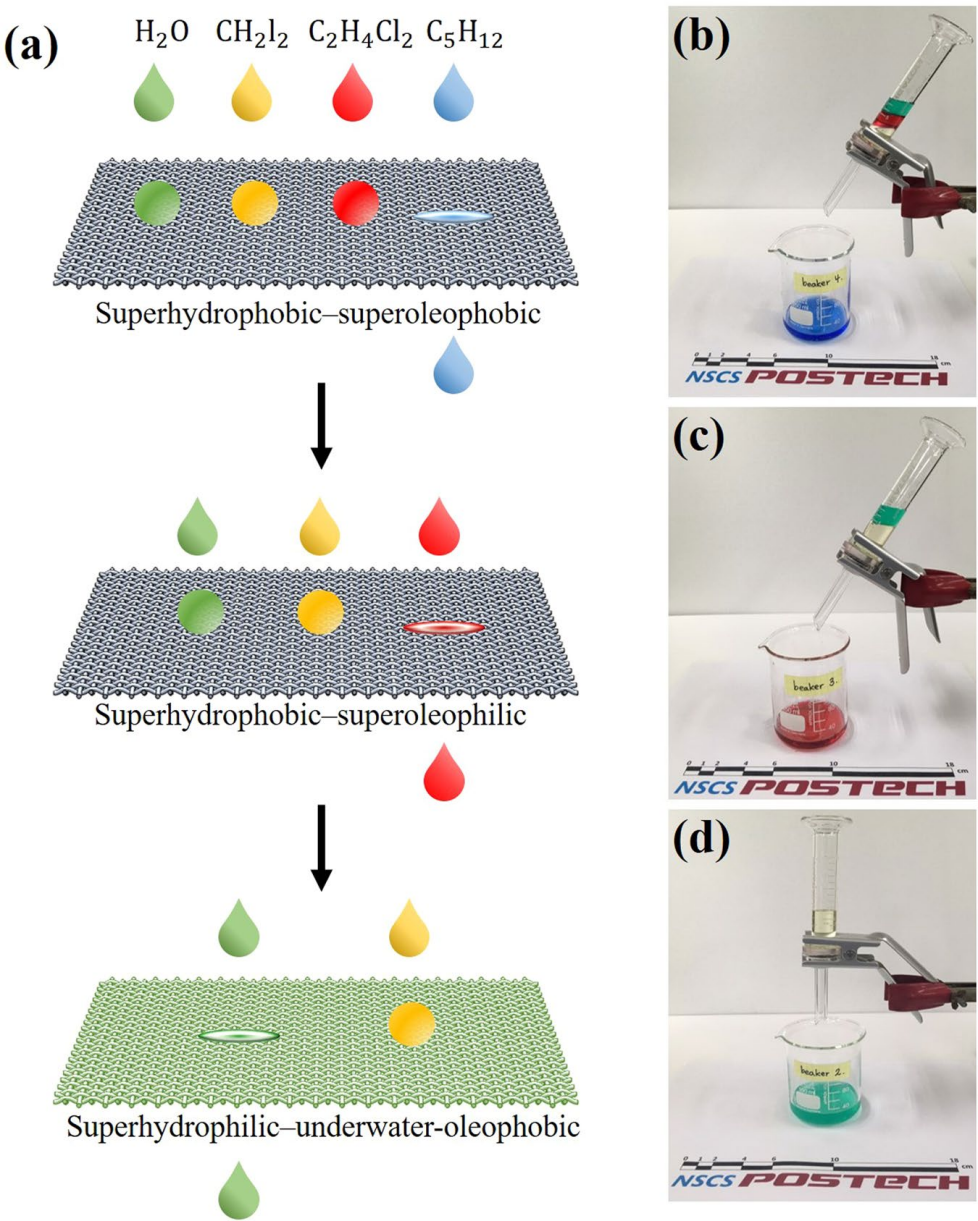
(**a**) Schematic illustration of multiphase liquid separation. (**b**–**d**) Separation process of multiphase liquid mixture consisting of water (H_2_O), diiodomethane (CH_2_I_2_), 1,2-dichloroethane (C_2_H_4_Cl_2_), and pentane (C_5_H_12_): (**b**) the separated H_2_O was collected and no other liquids were observed; (**c**) C_2_H_4_Cl_2_ was separated from the newly prepared H_2_O—CH_2_I_2_—C_2_H_4_Cl_2_ mixture; (**d**) after addition of a small quantity of water to the mesh, the separated CH_2_I_2_ was collected from the newly prepared H_2_O–CH_2_I_2_ mixture.

Figure 4(a–d) present the multiphase liquid-separation process. The liquids used in the separation test were water, diiodomethane, 1,2-dichloroethane, and pentane. When the mixture of liquids was poured over the superoleophobic mesh, water, diiodomethane, and 1,2-dichloroethane were retained, while the pentane component passed through. Thereafter, with selective permeation of 1,2-dichloroethane through the superoleophilic mesh, the residual 1,2-dichloroethane was separated from the retained water and diiodomethane. After that, water passed through the underwater-oleophobic mesh, but diiodomethane was blocked. No immiscible liquid was observed after the complete sequence of filtration processes. Because the liquids used in the test can be replaced with other liquids, the fabricated mesh can be used to sequentially separate various mixtures of four immiscible liquids.

**Figure 5:**
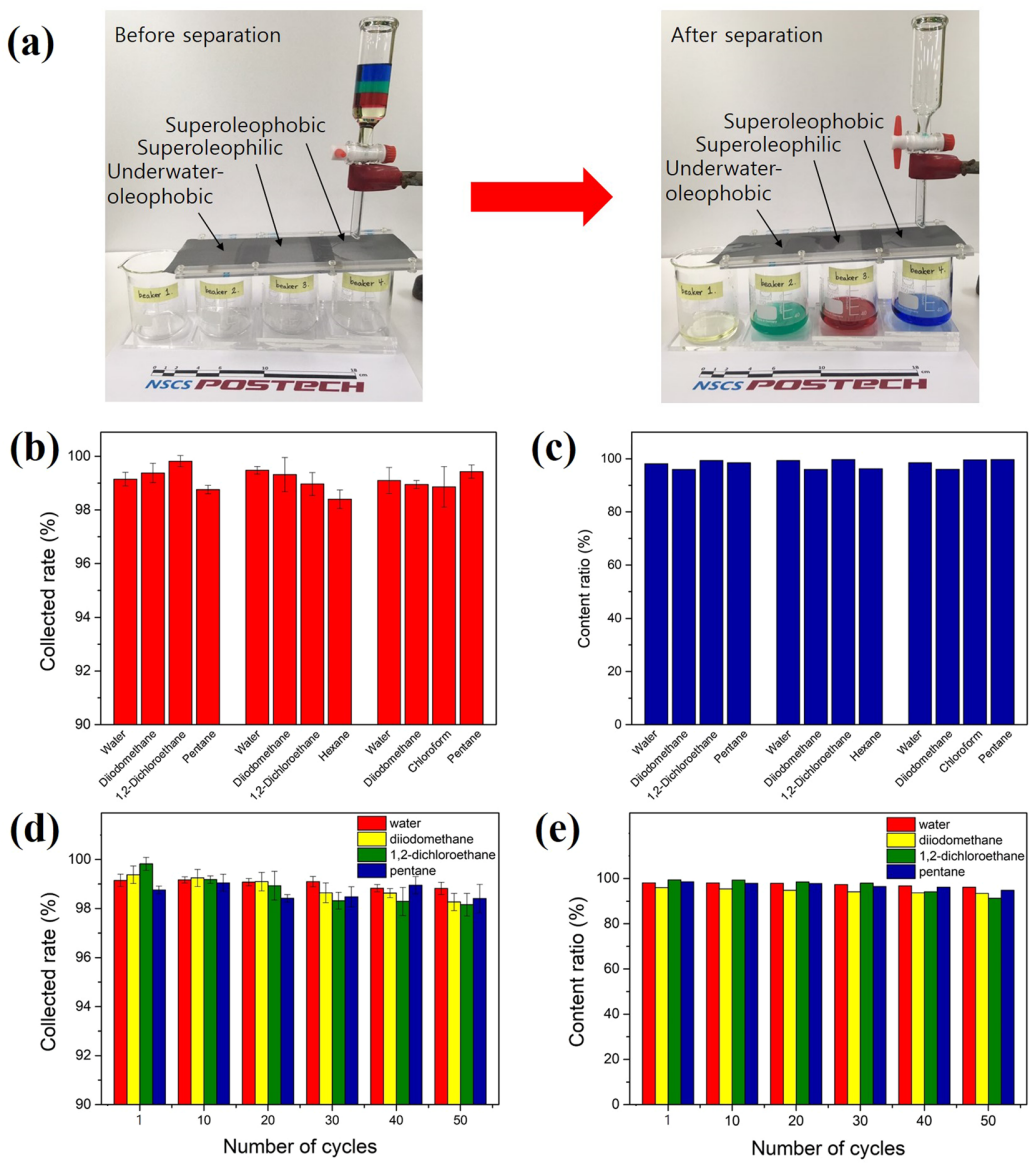
(**a**) Separation of various liquid mixtures; the liquid mixtures consisting of diiodomethane (with originally yellow), 1,2-dichloroethane (with red dye), water (with green dye), and pentane (with blue dye) were effectively separated by the as-prepared meshes. (**b**) Collection rate and (**c**) content ratio of various liquids separated by 50 mesh. (**d**) Collection rate and (**e**) content ratio of liquids that were separated from the multiphase liquid mixture versus the cycle number.

As shown in Fig. 5(a), the multiphase liquid-separation system used in our present work was quite simple. Before the liquid mixture was poured over the first mesh, the underwater-oleophobic mesh was pre-wetted with water, which was an essential step for successful separation. The liquids used in the separation experiments were pentane (with blue dye), 1,2-dichloroethane (with red dye), water (with green dye), and diiodomethane (natural yellowish colour). When the liquid mixture was poured into the separation system, the constituent liquids quickly passed through each mesh and collected in the accompanying beaker. The collection rate of each organic liquid mixture was calculated by the liquid collection rate coefficient (*η*, measured in %), according to following equation^26,27^:

$$\eta \,( \% )=\frac{{m}_{{\rm{a}}}}{{m}_{{\rm{b}}}}\times 100,$$

where *η* is the collection rate, *m*_a_ is the mass of the liquid before separation, and *m*_b_ is the mass of the liquid after separation. In order to more accurately evaluate the separation ability of the process, the content ratio of each collected liquid was measured. Separation experiments were performed using the 50, 100 and 200 meshes to demonstrate the separation efficiency according to mesh pore size. Supplementary Fig. S3 shows the collection rate and content ratio of the meshes with various pore sizes. It is clear that a mesh with a smaller pore size had a higher separation efficiency. Smaller pore sizes, however, are weak against physical forces, but the resulting increase in separation efficiency of a mesh is negligible. To verify the separation efficiency of the prepared filter, various organic liquid mixtures were prepared and separated using the same process. The first mixture consisted of water, diiodomethane, 1,2-dichloroethane, and pentane; the second mixture consisted of water, diiodomethane, 1,2-dichloroethane, and hexane; and the third mixture consisted of water, diiodomethane, chloroform, and pentane. Figure 5(b,c) show the collection rate and content ratio for separation of the four mixtures using the underwater-oleophobic, superoleophilic, and superoleophobic meshes, which all showed collection rates of >98% and content ratios of >96%. To evaluate the reusability of the prepared meshes, the efficiency of multiphase liquid separation was also investigated according to the number of separation cycles. The results in Fig. 5(d,e) show that the prepared meshes exhibited separation efficiencies that were not attenuated even after 50 cycles; the separation efficiencies of ≥92% suggest that the meshes were highly reusable and sufficiently stable.

**Figure 6:**
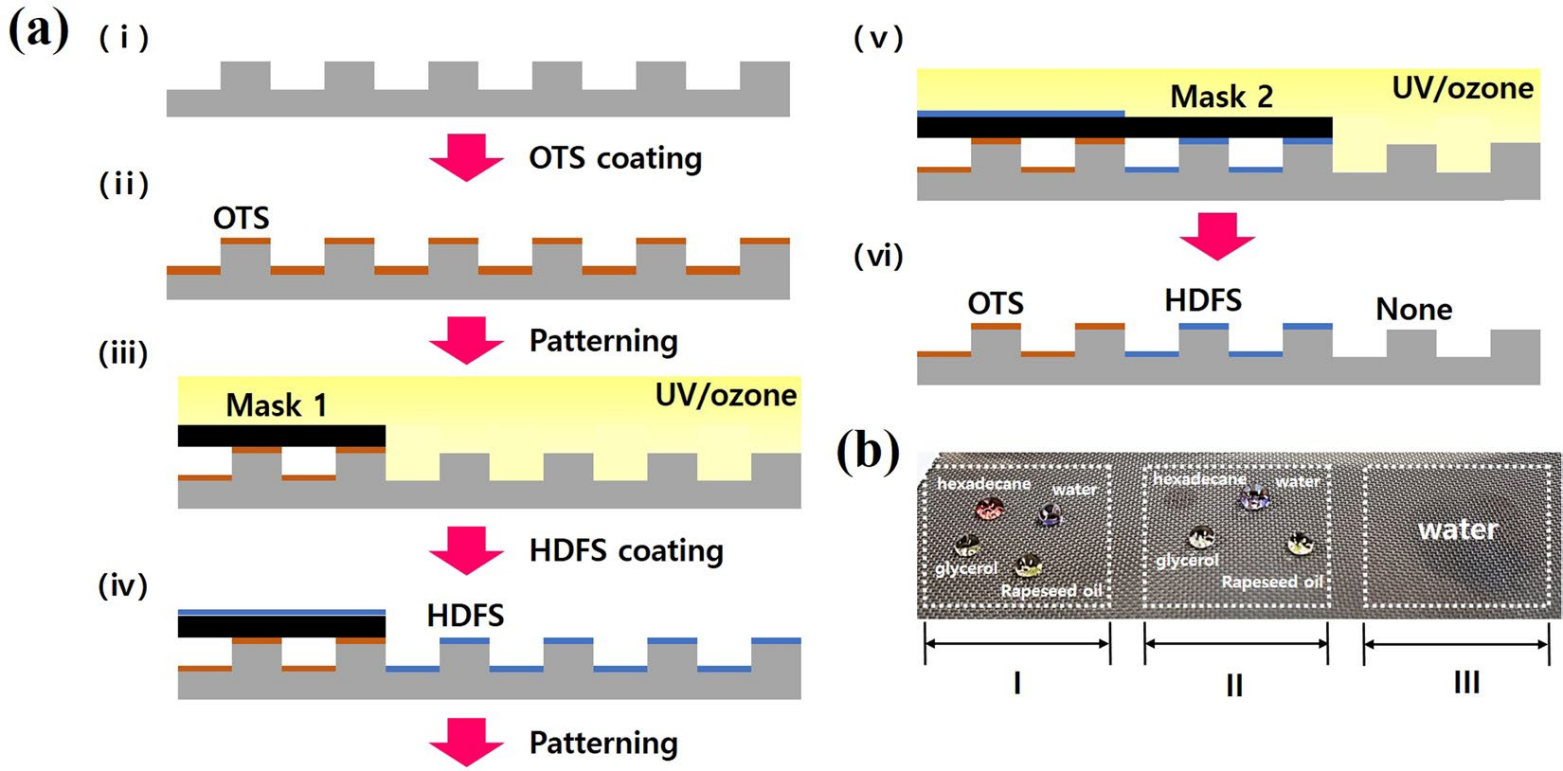
(**a**) Schematic of fabrication of surface with heterogeneous wettability pattern. (**b**) Optical image of the fabricated surface with heterogeneous surface energies.

In order to create a patterned surface with multiple wettability in one mesh, the surface was modified by an alkyltrichlorosilane coating and UV photopatterning, as shown in Fig. 6(a). The hierarchical microcube–nanohole structured mesh was first dipped in an OTS coating solution. The OTS-coated mesh was then exposed to UV light through a photomask. The alkyl chain of OTS at the irradiated sites was completely decomposed by photochemical reactor after 2 h of UV exposure^28–30^. HDFS coating and patterning were performed using similar procedures. Figure 6(b) shows an optical image of the developed patterned surface with heterogeneous surface energies. The surface consisted of an HDFS-coated area (superhydrophobic–superoleophobic) (I), an OTS-coated area (superhydrophobic–superoleophilic) (II), and an uncoated area (superhydrophilic–underwater-oleophobic) (III). Thus, if the coating materials on the hierarchical microcube–nanohole structured mesh were selected in terms of the desired wettability for the target solutions, a patterned surface with a wide range of wetting properties could be obtained. In addition, by simply changing the order of the patterns on the surface, the four liquid mixtures could be separated by only one mesh. All these results demonstrate that this system with underwater-oleophobic, superoleophobic, and superoleophilic meshes exhibited excellent separation efficiency and will contribute to the development of advanced multiphase liquid separation for practical applications.

## Discussion

A novel superhydrophilic–underwater-oleophobic mesh was successfully fabricated, and a multiphase liquid-separation system was fabricated using superhydrophilic–underwater-oleophobic, superhydrophobic–superoleophilic, and superhydrophobic–superoleophobic meshes for gravity-driven multiphase liquid separation. AFM images, EDS, contact-angle, and sliding-angle measurements demonstrated that these surfaces possessed the proper surface roughness and wettability required for efficient selective separation. The system showed high collection rates (≥98%) and high content ratios (≥96%) for various liquid mixtures. These results indicate that the fabricated meshes were chemically robust against various kinds of liquids that usually require filtration. In addition, we observed excellent collection rates (≥98%) and content ratios (≥92%) in 50 cycles of repeated separation. By adjusting the chemistry of the surface, we demonstrated that multiple liquids could be separated by one mesh whose patterned surface was modified to have heterogeneous wettability. Therefore, the present study is expected to contribute towards the development of advanced innovative liquid-separation systems with promising application in many practical situations.

## Materials and Methods

Aluminium meshes with a pore size of 300 µm (50 mesh) were used as the substrates. First, an aluminium mesh was dipped in 3 M HCl solution at 80 °C for 30 s to form microstructures. Then, the microstructured aluminium mesh was anodized in 0.3 M oxalic acid solution at 26 °C under a constant current of 2.5 A for 25 min using a circulator (Lab Companion, RW-0525G) to form nanostructures on the microstructures. After complete washing and drying of the fabricated aluminium mesh, the mesh was ready for use. Three meshes with the same structure were coated with water to impart superhydrophilic–oleophobic characteristics for the preparation of a superhydrophobic-superoleophilic coating with OTS and a superhydrophobic-superoleophobic coating with HDFS.

## Supplementary information

Supplementary information
