# Supplementary material for: Successive four-phase liquid separation using hierarchical microcube-nanohole structure and controlled surface wettability meshes
Source: Sci Rep. 2019 Apr 24;9:6503. doi: 10.1038/s41598-019-43003-1 (PMC6482191; doi:10.1038/s41598-019-43003-1)
Supplement: Supplementary file 1 — Supplementary information [file 41598_2019_43003_MOESM1_ESM.docx]

**Supplementary Information**

**Successive four-phase liquid separation using hierarchical microcube-nanohole structure and controlled surface wettability meshes**

Seeun Woo, Woonbong Hwang*

*Department of Mechanical Engineering, POSTECH, Pohang 37673, Republic of Korea*

*Corresponding author. whwang@postech.ac.kr

**Supplementary Figures**


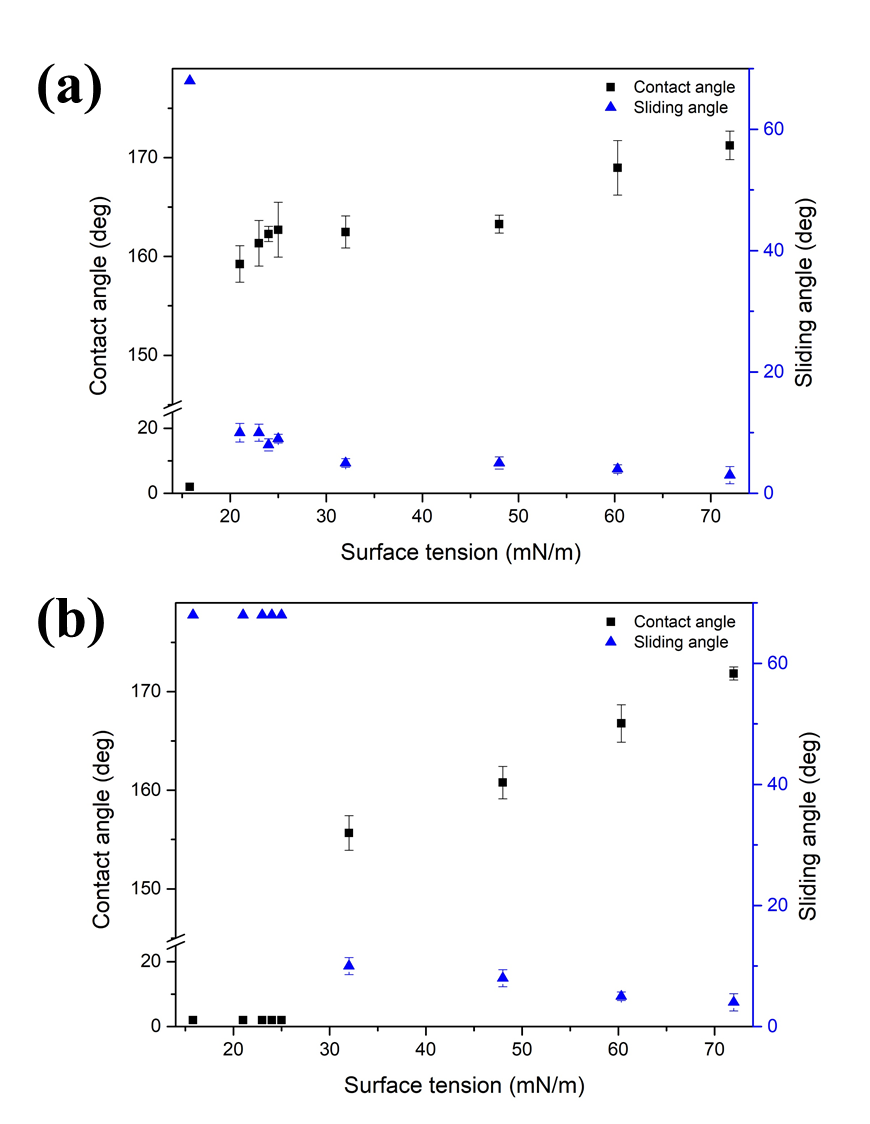


**Figure S1.** Graphs showing (**a**) contact angles and (**b**) sliding angles as functions of the surface tension of various liquids on superhydrophobic–superoleophobic and superhydrophobic–superoleophilic meshes.


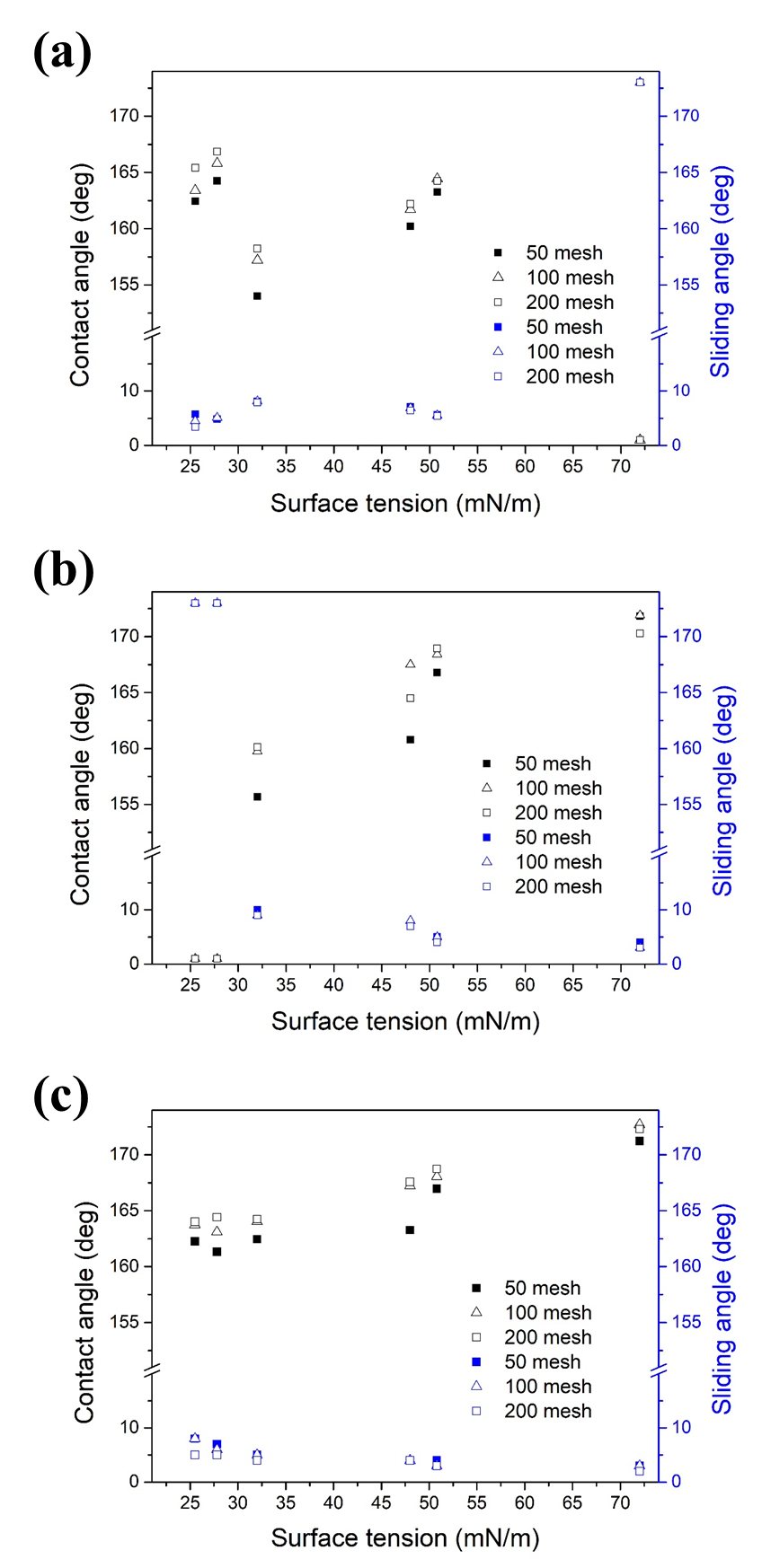


**Figure S2.** Graphs showing contact angles and sliding angles as functions of the surface tension of various liquids on superhydrophilic–underwater-olephobic, superhydrophobic–superoleophobic and superhydrophobic–superoleophilic 50, 100 and 200 meshes.


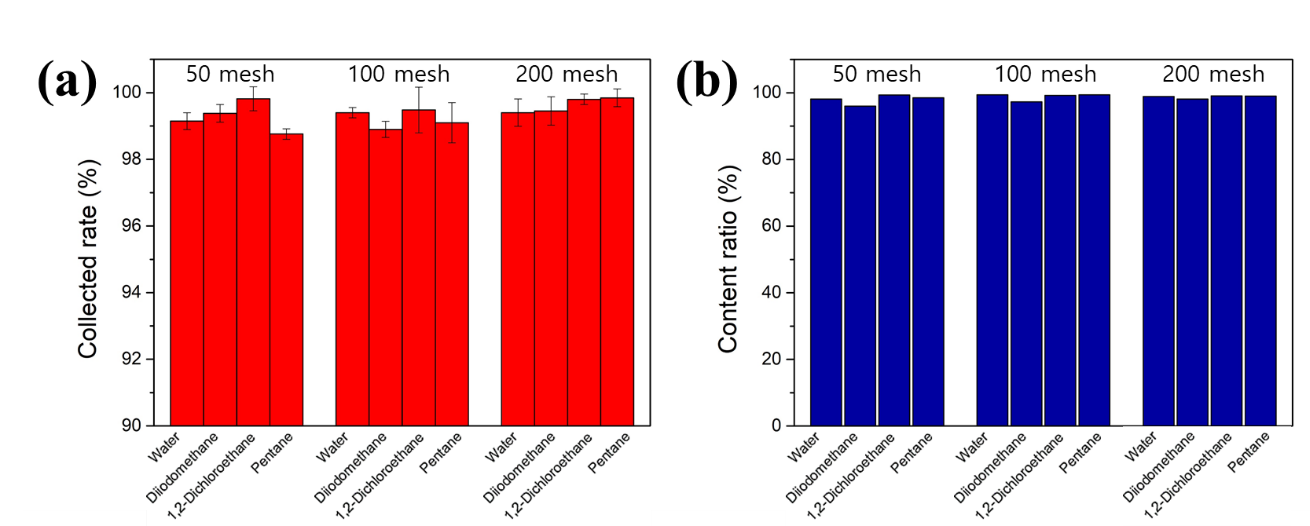


**Figure S3.** (a) Collection rate and (b) content ratio of the superhydrophilic–underwater-oleophobic, superhydrophobic–superoleophilic, and superhydrophobic–superoleophobic meshes as functions of pore size.
